# Urothelial Carcinoma With Divergent Glandular Differentiation

**DOI:** 10.7759/cureus.72603

**Published:** 2024-10-29

**Authors:** Hristo Popov, Andreya Kirilova, Kristina Naydenova, Ekaterina Softova, George S Stoyanov

**Affiliations:** 1 General and Clinical Pathology, Forensic Medicine and Deontology, Medical University of Varna, Varna, BGR; 2 Pathology, Individual Medical Diagnostic Laboratory City Lab, Varna, BGR; 3 Pathology, Multiprofile Hospital for Active Treatment, Shumen, BGR

**Keywords:** adenocarcinoma, divergent differentiation, malignancy recurrence, urinary bladder, urothelial carcinoma

## Abstract

Divergent differentiation in urothelial carcinoma is a challenging phenomenon that often makes the proper histopathological diagnosis a challenge. Conventional foci are often scarce in this subclass of urothelial carcinoma, and even if proper grossing and slide processing is performed, inexperienced pathologists can miss them. Furthermore, divergent differentiation in urothelial carcinoma is important to be recognized as such not only for the true diagnosis to be established and hence not increase the statistical incidence of rare neoplasms but also as divergent differentiation is one of the key factors in defining urothelial carcinomas as high-grade and hence crucial for proper patient management and treatment. Herein, we report a histopathological case of a male patient in his 60s who had been diagnosed with a high-grade urothelial carcinoma with submucosal involvement one year prior. Due to symptom recurrence, a follow-up endoscopy and resection of suspicious areas were performed to check for disease recurrence. Histopathology revealed a malignant neoplasm with histomorphological features of predominantly colonic-type adenocarcinoma and only a few areas with urothelial papillary morphology. Hence, divergent differentiation of the neoplasm was noted. Other than not falsely increasing the incidence of rare bladder malignancies, proper recognition of urothelial divergent differentiation is also important for excluding metastatic lesions to the bladder, as most of the diverging types of differentiation are common in neighboring organs or in tumor types that have pronounced metastatic potential.

## Introduction

Urothelial malignancies develop from the native transitional epithelium of the urinary system, also referred to as urothelium [1]. As such, these malignancies can develop throughout the urinary system; however, their most common site of occurrence is the urinary bladder [1]. Other casuistic locations of urothelial carcinoma development through other mechanisms are the female reproductive system, where they can develop in the uterine cervix and ovaries [2,3].

Urothelial carcinoma grading

The International Society of Uropathology (ISUP) and the World Health Organization (WHO) define urothelial malignancies as having two stages of differentiation: low and high grade, based on architectural and predominantly cytological features, which are poorly transcribed from previous grading schemes defining these malignancies as highly, intermediately, and poorly differentiated [4]. While some overlapping features exist, the previous grading schemes relied on architectural and cytological features. In contrast, in the current grading system, the grade is determined based on the degree of atypia (cytological and predominantly nuclear), with even minor foci of evident and easily identifiable nuclear size variation, hyperchromasia, nuclear membrane angulation, irregular contours, increased mitotic activity, atypical mitoses, as well as more moderate nuclei but scant and amphophilic cytoplasm defining tumors as high-grade [5,6]. At the same time, most of these would fall into moderately differentiated based on the previous grading scheme. These changes were introduced as they better correlated with disease response to treatment, recurrence, and progression.

Regardless of the degree of cytological atypia, two other key features that also define high-grade urothelial malignancies are the presence of histological subtypes and divergent differentiation.

Subtypes of urothelial carcinoma

Urothelial carcinoma subtypes are defined as those malignancies that exhibit a specific growth pattern or cellular features other than purely papillary growth. These subtypes include micropapillary carcinoma, where there is limited papillary growth with lack of fibrovascular cores, more often resembling tumor-cell clusters [7]; nested and large nested urothelial carcinoma where cytological bland tumor cells grow in small or larger invasive nests and can often be mistaken for Brunner nests [8,9]; tubular and microcystic type wherein the atypical urothelial cells, albeit with predominantly limited atypia grow in a gland-like tubular of cystic invasive pattern [10]; plasmocytoid urothelial carcinoma, where albeit significantly atypical urothelial cell, often with lack of tumor cell cohesion invade the bladder stroma and can often the mistaken for plasma and other inflammatory cells [11]; lipid-rich urothelial carcinoma present with optically empty, lipid-filled cytoplasmic vacuoles, often mimicking lipoclastic differentiation [12]; clear cell, also referred to as glycogen-rich urothelial carcinoma presents with pale to optically empty cytoplasm of the tumor cells, due to accumulation of glycogen, which can be proved on histology by means of special stains [13]; lymphoepithelioma-like urothelial carcinoma presents with significantly atypical cells, predominantly with a pale cytoplasm growing in clusters, often with syncytial appearance with significant lymphocytic infiltration in the stroma [14]; giant cell urothelial carcinoma present with frank bizarre, multinucleated and monstrous tumors cell, often lacking evident urothelial differentiation [15]; poorly differentiated urothelial carcinoma is similar to the giant cell one; however while the cells typically lack any evidence of urothelial differentiation both architecturally and cytoloigcally, there is a varying presence of bizarre and multinucleated cells of epithelial origin [16]; sarcomatoid urothelial carcinoma where the cell display frank sarcoma features - spindled and significantly atypical, this subtype is often admixed with other subtypes and diverging differentiation such as squamous, neuroendocrine and/or glandular [17].

Urothelial carcinomas with divergent differentiation

Divergent differentiation is defined as a urothelial neoplasm with a secondary type of evident architectural and cytological differentiation within the same tumor, often showing a divergent molecular profile. These include squamous differentiation, the most common one, accounting for around one-third of cases [18]. The second most common is glandular differentiation, wherein the tumor displays a frank adenocarcinoma component [18]. This divergent differentiation is often mistaken for lumina-like spaces forming within the urothelial papillae, a significantly more common occurrence. The presence of conventional urothelial carcinoma can help with the diagnosis in cases where it is unknown, especially in cases where it is known that the patient has adenocarcinoma in another location, to differentiate from adenocarcinoma metastatic spread. The two most exotic types of divergent differentiation allude to the embryonal origin and the rare occurrence of urothelial carcinoma in the female reproductive system: trophoblastic and Mullerian differentiation. In urothelial carcinomas with trophoblastic differentiation, the tumor displays a frank trophoblastic or syncytiotrophoblastic appearance, which can be confirmed via immunohistochemistry, and this differentiation must be distinguished from the giant-cell type of urothelial carcinoma. Further confirming the divergent nature of this cellular component is the fact that not only do some tumors display frank choriocarcinoma appearance, again necessitating the differential diagnosis of metastatic spread but also elevated serum levels of beta human choriogonadotropin [19]. Mullerian differentiation can often be viewed as controversial, as the separate entry of clear cell adenocarcinoma of the urinary tract can often display a urothelial component [20]; however, the growth patterns such as solid, tubulocystic, and papillary, together with predominantly hobnail-appearing cells that do not give positivity to urothelial markers on immunohistochemistry, sway opinions that this is a separate rare nosological unit rather than urothelial carcinoma with divergent differentiation [19-20].

As seen by the many types and forms of divergent differentiation and the common occurrence of histologically similar or identical malignancies throughout the body, diagnosing these high-grade malignancies is often dubious.

## Case presentation

**Figure 1:**
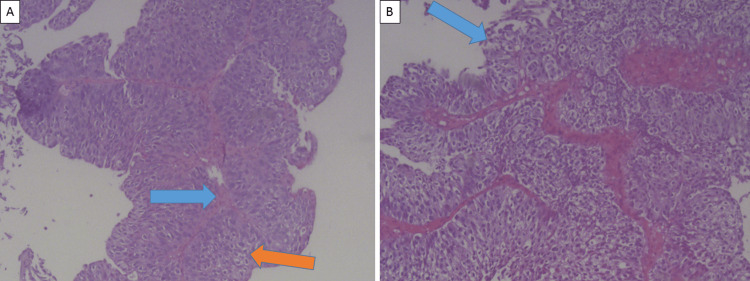
Histology of the resected specimen A: papillary structure with a fine fibrovascular core (blue arrow), covered with multiple layers of atypical urothelial cells with nuclear hyperchromasia and variation in size (orange arrow); B: branching and fusing papillae with atypical cells showing loss of polarity (blue arrow) Hematoxylin and eosin stain, original magnifications x100

Herein, we present a case report of a 66-year-old male patient. The patient initially presented to our institution one year prior with complaints of gross hematuria, dysuria, polyuria, and a decrease in urine stream. Previous medical history was significant for tobacco smoking for the previous 30 years, 40 pack years in total, and mild hypertension under adequate medication control for the previous 15 years. Based on his presenting symptoms and under suspicion of bladder malignancy, a bladder endoscopy was scheduled. The endoscopy showed a fine branching tumor formation in the posterior bladder's lower aspect, which was successfully excised. Histology revealed a fine branching and papillary tumor with fusing fibrovascular cores covered by multiple layers of atypical urothelial cells with loss of polarity, anisocytosis, and anisocariosis with foci of nuclear enlargement and hyperchromasia, focal vesicular nuclei and prominent nucleoli, focal atypical mitotic figures, and areas of invasion into the submucosa. Based on the histological depiction of the tumor, the diagnosis of a high-grade papillary urothelial carcinoma was established, and the tumor was staged as pT1 based on the presence of submucosal invasion (Figure 1). The patient was referred to an oncological committee, and intravesical treatment and monitoring were initiated as treatment.

The patient's current presentation was due to symptom recurrence and suspicion from the monitoring oncologist and urologist for disease recurrence and/or progression. Hence, a new bladder endoscopy was scheduled, which showed evidence of tumor recurrence in the initial topographic zone of the bladder, with the tumor having significantly fewer visible papillae and exhibiting a more flat growth pattern.

**Figure 2:**
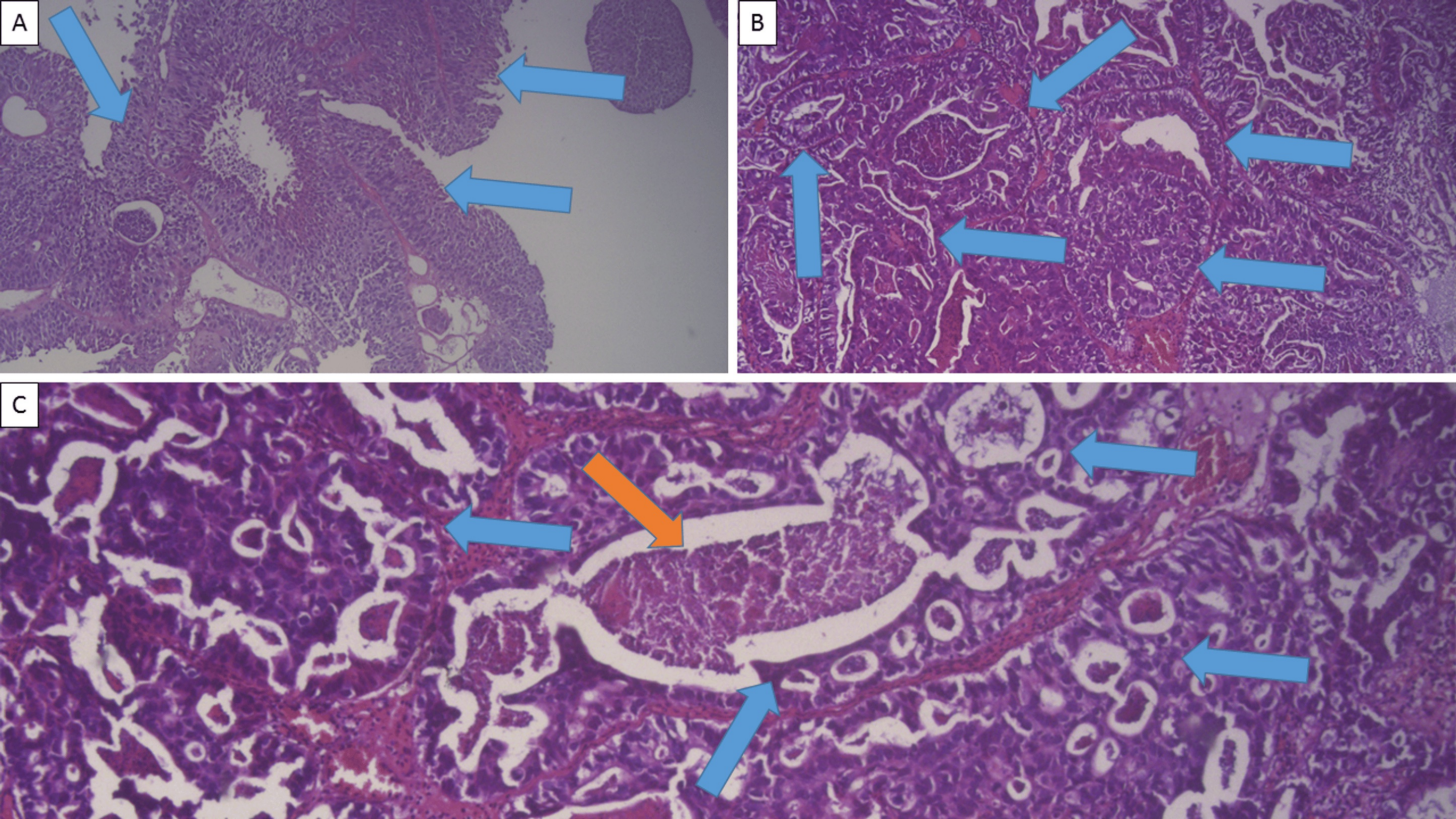
Histopathology of tumor recurrence A: papillary structures (blue arrows); B: nest with luminal formation (blue arrows); C: cribriform glands (blue arrows) with necrotic debris in luminal spaces (orange arrow) Hematoxylin and eosin stain, original magnification x100

Histopathology of the resected specimen showed focal finely branching and fusing papillary structures with atypical urothelial cells akin to the initial biopsy (Figure 2A). However, there were also scattered nests with cribriform lumina formations covered by a single layer of atypical cylindrical cells akin to colonocytes (Figure 2B). Some of the luminal spaces were filled with necrotic debris. Some of the glandular structures showed evident submucosal invasion without identifiable invasion within the smooth muscle component of the bladder wall. As the patient had no evidence of glandular malignancy elsewhere in the body, as well as a history of high-grade urothelial carcinoma and the presence of evident urothelial differentiation of the lesion, the diagnosis of recurrent high-grade urothelial carcinoma with new-onset divergent glandular differentiation was established and once again staged as pT1. Following the diagnosis, the patient was again referred to the oncological committee, and intravesical therapy was re-initiated. On follow-up, the patient is stable without new-onset complaints.

## Discussion

Divergent differentiation in urothelial carcinomas often leads to diagnostic dilemmas [21]. As the two most common types of divergent differentiation are squamous and glandular, and with the abundance of such malignancies throughout the body, especially within close proximity to the urinary system, the diagnosis is often clinicopathological and one of exclusion [18]. Current ISUP and WHO recommendations for the diagnosis of cases with extensive divergent differentiation recommend that in the presence of previous conventional urothelial carcinoma or the presence of any evidence of urothelial differentiation of the lesion, including areas of in situ carcinoma, the cases should be specified as urothelial in nature [22]. Detailed clinical history and additional evaluations and investigations also help exclude a malignancy of similar histology and different primary sites.

The diagnosis becomes even more difficult in cases with unclear or unavailable previous medical history, as despite rarely primary squamous and adenocarcinoma can also develop within the urinary bladder. One such condition is the aforementioned clear cell adenocarcinoma of the urinary tract, which can also be viewed as a urothelial carcinoma with divergent Mullerian differentiation [23]. Still, in this nosological unit, the histological findings are somewhat unique, and the clinical search for a primary is somewhat limited.

The rare cases of true urinary bladder adenocarcinoma require a more detailed search. We have previously published such a case of primary urinary bladder adenocarcinoma, with extremely similar histopathology akin to intestinal adenocarcinoma, which was associated with cystitis cystica et glandularis [24]. Furthermore, in such cases, neither immunohistochemical testing nor genetic testing are of any aid as both the true adenocarcinoma of the bladder, bladder adenocarcinoma metastasis, and urothelial carcinoma with divergent glandular differentiation present with no urothelial profile, apart from the urothelial component of tumors with divergent differentiation [25,26]. However, this is another aspect of the tricky diagnostic process, as they require an experienced endourologist to properly and completely excise the lesion, an experienced pathologist and laboratory technician to properly and fully process the gross specimen, as well as the pathologist to not miss even minuscule areas of urothelial differentiation on histology [25].

True urinary bladder adenocarcinomas rarely develop on their own, most often developing from fetal remnant tissue such as urachal adenocarcinoma or in the context of chronic inflammation with intestinal metaplasia [24,26]. As already depicted above, these lesions have no histological or molecular evidence of conventional urothelial differentiation and, on their own, represent a diverse group of rare malignancies [24].

## Conclusions

Divergent differentiation in urothelial carcinoma is a relatively rare occurrence that, on its own, designates these malignancies as high-grade. It is also challenging for the pathologist due to the myriad of possible metastatic lesions to the bladder, together with the often minuscule evidence of urothelial differentiation, sometimes only in the form of in situ urothelial carcinoma. While often in modern-day pathology, immunohistochemical and molecular analysis are key in defining the diagnosis, in such lesions, they are useless due to the diverging line of differentiation, losing all urothelial markers and acquiring those of the diverging line.
